# Understanding the emergence of bacterial pathogens in novel hosts

**DOI:** 10.1098/rstb.2018.0328

**Published:** 2019-08-12

**Authors:** Camille Bonneaud, Lucy A. Weinert, Bram Kuijper

**Affiliations:** 1 Centre for Ecology and Conservation, University of Exeter, Penryn TR10 9FE, UK; 2 Department of Veterinary Medicine, University of Cambridge, Cambridge CB3 0ES, UK

**Keywords:** emerging infectious disease, host range, phenotypic plasticity, specialization, spillover, host shift

## Abstract

Our understanding of the ecological and evolutionary context of novel infections is largely based on viral diseases, even though bacterial pathogens may display key differences in the processes underlying their emergence. For instance, host-shift speciation, in which the jump of a pathogen into a novel host species is followed by the specialization on that host and the loss of infectivity of previous host(s), is commonly observed in viruses, but less often in bacteria. Here, we suggest that the extent to which pathogens evolve host generalism or specialism following a jump into a novel host will depend on their level of adaptation to dealing with different environments, their rates of molecular evolution and their ability to recombine. We then explore these hypotheses using a formal model and show that the high levels of phenotypic plasticity, low rates of evolution and the ability to recombine typical of bacterial pathogens should reduce their propensity to specialize on novel hosts. Novel bacterial infections may therefore be more likely to result in transient spillovers or increased host ranges than in host shifts. Finally, consistent with our predictions, we show that, in two unusual cases of contemporary bacterial host shifts, the bacterial pathogens both have small genomes and rapid rates of substitution. Further tests are required across a greater number of emerging pathogens to assess the validity of our hypotheses.

This article is part of the theme issue ‘Dynamic and integrative approaches to understanding pathogen spillover’.

## 1. Introduction

Our ability to predict and prevent novel outbreaks of infectious pathogens hinges on a thorough understanding of the conditions favouring their emergence, from their initial jump through to their adaption to the novel host environment [1]. Yet, because outbreaks of current concern are mainly viral, our understanding of the ecological and evolutionary context of pathogen emergence stems largely from viral infections [2,3]. Whether we can generalize these findings to other types of pathogens remains to be carefully thought through, particularly given substantial differences in ecology and life history between viruses, protists, fungi and bacteria.

The successful emergence of a pathogen following a jump into a novel host species can occur though two mechanisms [4]: (i) host shift, in which the jump is followed by specialization on the novel host and loss of infectivity of the original donor host(s) [5,6]; and (ii) increased host range, in which the jump expands the number of host species that the pathogen can infect. Host shift speciation is often observed in plant viruses, fungi and parasitoids, and in animal viruses, but may not be as common in bacterial pathogens [7]. Indeed, a review of host–symbiont cophylogenies revealed that, for studies in which a process could be inferred, viruses were most likely to have speciated as a result of host shift in all 13 cases reported, while bacteria were most likely to have done so in six cases, with the remaining eight cases of bacterial speciation occurring alongside host speciation (i.e. through cospeciation) [7]. Thus, predicting the epidemiological and evolutionary consequences of bacterial jumps into novel hosts will require a better understanding of the processes that maintain host generalism and/or constrain the evolution of host specialization.

Here, we propose three non-mutually exclusive hypotheses to explain why novel bacterial pathogens may be less likely to specialize on their novel hosts. Specifically, we hypothesize that a pathogen's adaptation to dealing with different environments, rate of molecular evolution and ability to recombine will affect its propensity for host specialization. We then use a modelling approach to generate predictions as to how these factors affect evolutionary diversification when pathogens are exposed to novel hosts. Finally, we examine two rare cases of bacterial host shifts to identify what may make these systems unusual.

## 2. Factors influencing host specialization

### (a) Adaptation to environmental variation

Unlike viruses that reside mainly within host cells, bacteria can exploit a range of different habitats, sometimes during their life cycle: they can be found in the host environment either inside or outside cells (including in the circulatory and gastrointestinal system), and in the external environment [8]. Even within these broad categories of lifestyle, there appears to be some variation [9]: bacteria commonly thought of as extracellular, such as *Bacillus anthracis*, can be found intra-cellularly *in vivo* [10], while intracellular ones, such as *Legionella pneumophila*, are able survive and replicate in the environment outside of a host, such as in water or food [11]. Other types of bacteria still, including *Yersinia pestis*, can readily invade and replicate within different types of host cells and in the extracellular environment [12]. The plant pathogen *Pseudomonas syringae* can survive daily and seasonal climatic changes in temperature, humidity, ultraviolet light and moisture on the surface of leaves while also being capable of penetrating plant tissues and colonizing the intercellular apoplast space [13]. This ability to deal with environmental variation is likely to be owing, in part, to their limited reliance on the host replicative and metabolic machinery, as well as to an ability to generate rapid phenotypic variation, such as through phenotypic plasticity. For example, one mediating mechanism of plasticity known to occur in bacteria is a process termed ‘phase variation’, which randomly and reversibly switches gene expression [14]. Although this form of plasticity does not occur in response to specific environmental cues, it can underlie the progression of bacterial infections from asymptomatic to invasive, and drive their persistence in different host tissues [15]. Either way, if bacteria have evolved plasticity to rapidly deal with environmental variation, then genetic adaptation giving rise to divergence and specialization in the new host may be constrained, particularly if losing the ability to infect other hosts is costly [16]. One prediction from this hypothesis is that bacterial pathogens that are more opportunistic and able to exploit a larger number of environments will be less likely to evolve host specialization than obligate bacterial pathogens of just one or a few hosts.

### (b) Rate of evolution

A second hypothesis for why bacteria may be less likely to emerge through host shifts has to do with their rate of evolution. While bacteria and viruses may have a comparable number of mutations per genome per generation [17], viruses have higher census populations and so will contain more mutations per founding inoculum. In addition, the smaller genome sizes of viruses mean that any new mutation is likely to have a larger functional effect (as evidenced by the greater skewed distribution of fitness effects of new mutations in viruses) [18,19]. The typically high evolutionary rate of viruses is thought to drive their rapid adaptation to novel host environments, potentially to the point where the evolution of high host-specificity makes them prisoners of their hosts [20]. In contrast, the reduced rate of functional evolution of bacterial pathogens may limit (but not prevent) their ability to rapidly adapt and specialize on novel host environments. Bacterial infections may therefore more often result either in transient spillovers destined for eventual extinction in the novel host, or in increased host range if slower evolution occurs on the backdrop of frequent contacts between donor and novel host species. One prediction from the hypothesis of a role of molecular evolution in the propensity to host shift is that the likelihood of host specialization should increase with increasing mutation rates and decreasing genome sizes. Another prediction is that bacterial pathogens causing acute infections in their novel host should be less likely to evolve to become host specialists than those causing more chronic infections, because of the reduced amount of time to adapt and speciate in the former.

### (c) Recombination

Finally, the mechanisms by which bacteria generate genetic diversity itself may make it less likely for them to shift hosts. Recombination between bacterial populations, which allows the acquisition of novel genetic elements is, in general, thought to play a more important role in bacterial evolution than *de novo* mutations [21]. On the one hand, recombination could allow for faster host adaptation, as suggested by the emergence of *Xylella fastidiosa* in Mulberry, which was accompanied by a large recombination event [22]. On the other hand, the ability to acquire or lose genetic elements in accessory components of the genomes, while conserving an intact core genome, could be a key reason why bacterial pathogens are able to exploit a range of different environments rapidly and even simultaneously [23], without necessarily losing the ability to infect previous hosts. For example, variants of *Salmonella enterica* serovar Agona obtained over several outbreaks, sporadic infections and from the environment, displayed little changes in their core genomes, but wide variation in the accessory components of their genome as a result of the loss and gain of bacteriophages, plasmids and integrative conjugate elements [24]. Similarly, both *B. anthracis*, the causal agent of anthrax, and *Bacillus thuringiensis*, a pathogen of insects [25], are not only closely related to *Bacillus cereus*, a common soil bacterium, but they will act like *B. cereus* in the absence of plasmids (specifically: two virulence-determining plasmids in *B. anthracis* and one toxin-encoding plasmid in *B. thuringiensis*) [26,27]. In fact, the acquisition and loss of these mobile genetic elements are thought to be responsible for the divergence and reversion, respectively, of these bacterial pathogens from the ancestral, plasmid-free *B. cereus* [28]. In support for a role of plasmids in the maintenance of some level of host generalism, it is worth noting that the closely related, but mosquito-specialist bacterial pathogen, *Bacillus sphaericus*, has toxin-encoding genes distributed across its chromosome rather than on its plasmid [29]. The hypothesis of a role of recombination in the maintenance of host generalism therefore leads to the prediction that the acquisition of novel genetic elements from the environment may drive rapid adaptation to the novel host without a loss of infectivity of donor host(s).

## 3. Modelling the emergence of novel bacterial pathogens

Models of disease emergence typically focus on the probability of invasion by novel pathogens (see [30] for a review), while fewer predictions exist about whether these pathogens subsequently evolve to specialize or not on the novel host. Here we explore the hypotheses outlined above and generate specific predictions as to how the ability of pathogens to deal with environmental variability, their rate of molecular evolution and ability to recombine affect their tendency to evolve host specialization. We do this by building on existing studies that model the evolution of specialism versus generalism in virulence (e.g. [16]) and by tracking the evolution of an emerging pathogen in a population of two hosts [31]. As have previous multi-host models of virulence [31,32], we use an SI-model (susceptible–infected [33]) and describe the instantaneous changes in densities *S*_a_ and *S*_n_ of ancestral (donor) and novel hosts who are susceptible, and in densities *I*_a_ and *I*_n_ of ancestral and novel hosts who are infected, as:

3.1 $$\frac{dS_{a}}{dt}=\lambda (1-p)-\left(\delta_{a}+\underset{i\in \{a,n\}}{\sum }\beta_{ia}I_{i}\right)S_{a},$$

3.2 $$\frac{dS_{n}}{dt}=\lambda p-\left(\delta_{n}+\underset{i\in \{a,n\}}{\sum }\beta_{in}I_{i}\right)S_{n},$$

3.3 $$\frac{dI_{a}}{dt}=S_{a}\underset{\;j\in \{a,n\}}{\sum }\beta_{\;ja}I_{j}-(\delta_{a}+\alpha_{a}+\gamma_{a})\;I_{a}$$

3.4 $$\;\mathrm{and}\;\;\frac{dI_{n}}{dt}=S_{n}\underset{\;j\in \{a,n\}}{\sum }\beta_{\;jn}I_{j}-(\delta_{n}+\alpha_{n}+\gamma_{n})\;I_{n},$$

where the immigration rate of susceptible hosts (through either dispersal or fecundity) is given by *λ* and *p* reflects the proportion of immigrant hosts that are novel hosts. Next, *δ*_a_ and *δ*_n_ reflect the intrinsic mortality rates of ancestral and novel hosts. The expected number of secondary infections of hosts of type *j* per infected host of type *i* is given by *β_ij_ I_i_*, where *β_ij_* is the transmission rate from one individual host (of type *i*) to the next host (of type *j*) (see equation (3.5) below). Next, *α*_a_ and *α*_n_ reflect the virulence-induced mortality rates of ancestral and novel hosts, while *γ*_a_ and *γ*_n_ reflect the rates at which pathogens are cleared from ancestral and novel hosts.

As in the majority of models on the evolution of virulence (e.g. [32]), we assume that the rate of transmission of the pathogen from an infected host of type *i* to susceptible hosts of type *j* is given by *β_ij_* = *π_i_*(*v*)*ϕ_ij_*, where *π_i_*(*v*) is the production of pathogen propagules in host type *i* as a function of virulence *v*, and *ϕ_ij_* is a parameter that specifies the rate of transmission of pathogen propagules from host type *i* to host type *j*. We assume that the production of pathogen propagules increases in a decelerating fashion with pathogen virulence. Hence, in the ancestral host, we assume *π*_a_[*v*] = *v*/(*τ* + *v*) and, in the novel host, *π*_n_[*v*] = *π*_a_[(1 − *ρ*)*v*], where *τ* reflects the amount of virulence at which propagule production *π_i_*(*v*) is half of its maximum and *ρ* reflects how strongly the propagule production function diverges between ancestral and novel hosts. While increased virulence thus results in increased propagule production, it also reduces host mortality *α_i_* in ancestral and novel hosts according to *α*_a_ = *v* and *α*_n_ = (1 − *ρ*)*v* respectively.

Crucially, we then assume that pathogen virulence *v* is given by the reaction norm [34,35]:

3.5 $$v=a+b\varepsilon_{i},$$

where *a* is the genetic elevation, reflecting the amount of virulence that is expressed by the pathogen regardless of the host in which it resides. Following standard models on the evolution of virulence, we assume that *a* evolves through a successive number of mutations of small effect. Next, *b* is the pathogen's reaction norm slope, reflecting how virulence is plastically modulated dependent on cues within the host environment *ɛ_i_*. Using this formulation, we first assess how increased phenotypic flexibility (i.e. phenotypic plasticity) associated with bacterial lifestyles affects the propensity for pathogens to evolve to specialization after a jump into a novel host. Indeed, although it is well-known that the association between host specialization and phenotypic plasticity is negative (as host specialization and plasticity are two alternative evolutionary outcomes to adapt to a multi-host environment [16,36]), the exact shape of this relationship is still poorly understood. Specifically, we calculate whether disruptive selection results in evolutionary branching [37] of the genetic value for virulence *a* (equation (3.5)) as we vary the degree of phenotypic plasticity (measured as the reaction norm *b*; here a parameter) and the contact rate *ϕ*_an_ between the two host types (see electronic supplementary material). Electronic supplementary material, figure S1 shows that host shifts occur in pathogens with high amounts of phenotypic plasticity only when contact rates between the two hosts are extremely low. As soon as contact rates increase, however, even slight levels of phenotypic plasticity preclude the evolution of host specialization.

**Figure 1.**
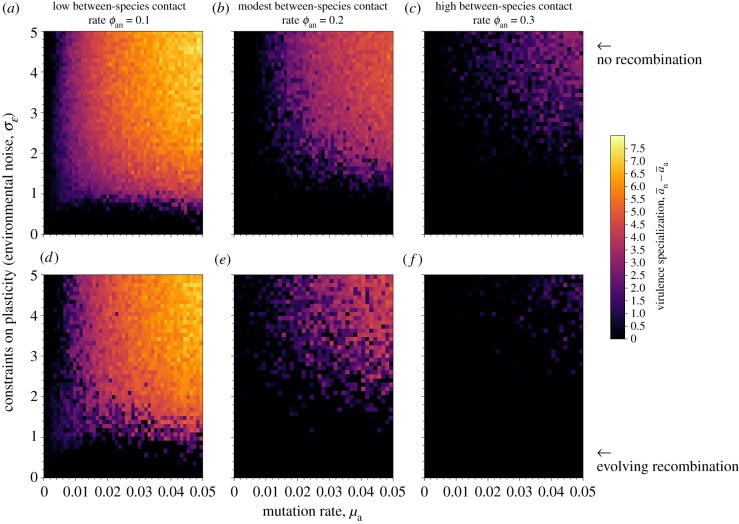
Host specialization by pathogens when recombination is absent (*a*–*c*) or evolving (*d*–*f*) for various levels of between host contact rates. Host specialization is favoured when recombination is absent (top row), plasticity is constrained (e.g. through an increasingly unpredictable environment) and mutation rates are high. Pathogen specialization is measured by taking the difference between average genetic values in novel and ancestral hosts ${\bar{a}}_{n}-{\bar{a}}_{a}$. Yellow indicates values of high host specialism and black values of high host generalism. See electronic supplementary material for parameter values.

We subsequently build on these analytical insights by using stochastic Gillespie simulations of the model in equations (3.1)–(3.5), where virulence *v_i_* in host species *i* is an additive function of: (i) evolving genetic loci *a* (mutating at rate *μ*) that reflect the baseline level of virulence across all environments; and (ii) evolving plasticity as a reaction norm slope *b* in response to a cue *ɛ_i_* that informs the pathogen about the host environment it lives in. To assess the role of constraints on plasticity, we assume that the environmental cue received by the pathogen in host *i* contains a certain level of white noise (distributed with mean 0 and standard deviation *σ_ɛ_*). Increasing levels of noise (i.e. larger values of *σ_ɛ_*) reduce the information content of the cue and hence disfavour the evolution of phenotypic plasticity. In addition, we assume that pathogens exchange loci *a* and *b* with other pathogens that reside within the same host species at an evolving recombination rate *h*. Figure 1 depicts the evolved levels of host specialization after 10^8^ timesteps for populations where recombination is absent (top row) and evolving (bottom row).

Figure 1 shows that bacterial features, such as: (i) a high flexibility of dealing with different environments (i.e. phenotypic plasticity, occurring when *σ_ɛ_* is low); (ii) a low mutation rate relative to viruses; and (iii) recombination, all reduce the prevalence of host specialization, ultimately resulting instead in pathogens with a broad host range. That the evolution of plasticity undermines pathogen specialization is well known (reviewed in [16]), yet other pathogen-specific characteristics such as mutation rates and recombination have received much less interest in the context of host specialization. Increased mutation rates enhance specialization as they allow for the rapid successions of host-specific mutations to kick in, thus preventing premature pathogen extinction in the novel host (see also [38] for the influence of mutation on specialization in the context of animal personalities). By contrast, recombination increases admixture between different pathogen lineages, thus precluding specialization. We find that recombination is strongly selected against and host specialization prevails only when selection favours high specialization as a result of low between-host contact rates (e.g. *ϕ*_an_ = 0.1 in figure 1*d*).

## 4. Case studies of bacterial host shifts

### (a) Staphylococcus aureus

This opportunistic bacterial pathogen of humans lives as a commensal within the nasal cavity, but can also cause soft tissue abscesses and a range of serious invasive diseases [39,40]. It is one of the leading causes of hospital-acquired infections, although infections outside of hospitals have increased in frequency in the past decade [41]. *Staphylococcus aureus* is, however, able to infect more than just humans and has been isolated from a range of domestic hosts, such as cows, sheep, goats, pigs and chickens, and wild hosts, such as rodents, non-human primates and bats [42–44]. Despite this apparent broad host range, there is evidence of some specialization of individual strains or clones on specific hosts [45–47].

There have been three documented cases of host shifts of *S. aureus* followed by pandemic emergence and adaptation to the new host [46,48,49]. The first case involves the clonal complex 5 (CC5) lineage, which emerged in poultry in the 1970s and subsequently spread globally to cause lameness in broiler chicken flocks. A genome analysis of poultry strains of CC5 showed that many human-related virulence genes were present but inactivated, lending support to phylogenetic evidence that the emergence was linked to a shift from humans [48]. The poultry CC5 isolates also acquired a novel mobile element that appears to confer protection against avian heterophils, suggesting a rapid adaptation to chickens. The second case involves the strain type 121 (ST121), which emerged in farmed rabbits in Europe following a jump from humans [49]*.* Adaptation to the novel rabbit host was associated with just one single nucleotide polymorphism. The third known case involves the transmission of the CC97 strain from livestock into humans. In fact, phylogenetic comparisons of human lineages with other livestock CC97 showed two independent jumps into humans from the major bovine *S. aureus* complex, one of which has now emerged globally [46]. Human isolates of CC97 have since increased over 10-fold during 5 years in Denmark [46].

The relatively high number of host shifts involving *S. aureus* is unusual, but may be explained by the fact that this bacterium has a smaller than average genome and a high molecular clock rate [50], both of which might facilitate the evolution of host specialization (see above). While we have so far focused on a discussion of the pathogen features that may have influenced speciation in the novel hosts, this system also points to a role of two ecological factors that may have facilitated the jumps. First, time-scaled phylogenetic analyses reveal that the known host shift of this bacterium all date to within the past 100 years (but see [51,52] for evidence of more ancient host shifts), thus suggesting a role for the globalization of agriculture in bacterial emergence. Second, *S. aureus* has overpowered virtually every antibiotic that has been developed [41], suggesting that we may now be seeing host jumps that we would have previously prevented through treatment.

### (b) Mycoplasma gallisepticum

This economically-important bacterial pathogen of poultry was detected for the first time in a wild North American passerine, the house finch (*Haemorhous mexicanus*), in 1994 [53,54]. The epidemic of severe conjunctivitis that ensued in house finches resulted from a single jump from poultry. Indeed, a phylogenomic study of 16 strains of *M. gallisepticum* obtained from poultry and from house finches in the first 12 years of the epidemic revealed that all finch strains descended from a single ancestor and coalesced roughly around the time of detected outbreak in the field [55].

This single jump from poultry was followed by the rapid divergence and specialization on the novel finch host. House finch strains of *M. gallisepticum* appear unable to infect the original poultry host: no transmission was detected when naturally infected house finches were housed with chickens but not allowed direct contact [56]. Furthermore, chickens finally seroconverted only after prolonged direct contact (more than 10 weeks) with infected house finches, but *M. gallisepticum* was actually detectable only in 20–30% of chickens. Similar results were obtained when *M. gallisepticum* was directly inoculated into chicken using the natural transmission route of the pathogen in house finches (i.e. eye drops) (M. Staley and C. Bonneaud, personal communication). In this case, the house finch strain displayed a significantly reduced ability to cause infection than the virulent poultry strain Rlow, with less than 15% of chickens infected with the house finch strain showing infection 14 days post-infection and only a single individual out of 22 displaying very mild symptoms.

Nevertheless, the host shift into house finches appears to have been a rare event. *Mycoplasma gallisepticum* has been detected in a number of other wild passerines, but these infections consist only of spillovers, with the bacterium being unable to persist and transmit within these other host species [57]. Similarly, spillovers from poultry into house finches may occur equally frequently; that one wild-caught, asymptomatic house finch was found infected with a poultry strain despite sporadic and random sampling, is startling. In other words, the single host shift of *M. gallisepticum* into house finches occurred against a backdrop of frequent spillovers of the bacterial pathogen into other host species.

So what unusual quality of *M. gallisepticum* allowed it to evolve specialization on house finches? Similarly to *S. aureus*, *M. gallisepticum* displays a small genome of approximately 1 Mbp and an unusually high rate of nucleotide substitution [55], both of which may have driven rapid host specialization. But this system also points to factors that may have played a role in successful pathogen emergence. First, we know the jump into house finches was accompanied by a significant loss of CRISPR repeat diversity followed by the gradual complete loss of CRISPR function [55]. This could be explained by a loss of bacteriophage pathogens as *M. gallisepticum* jumped into house finches; whether this would have been a cause or a consequence of the jump is unfortunately untestable, but we can at least hypothesize that it resulted in a selection release that allowed the bacterium to respond to other selection pressures (i.e. the novel host). Second, it is conceivable that the conditions experienced by the emerging lineage when it was still in the original poultry host somehow ‘pre-adapted’ it to deal with the novel host environment. Previous experimental work has shown that poultry *M. gallisepticum* cannot successfully infect house finches, such that mutations arising in the poultry host would have been necessary for pathogen emergence in house finches [58]. What those mutations were and what phenotypic consequences they might have had still need to be investigated. Either way, the shift of *M. gallisepticum* into house finches appears to have been an unlikely event driven by a rare set of circumstances.

## 5. Conclusion

Bacterial pathogen emergence following a jump into a novel host appears to rarely give rise to the evolution of host specialization that is characteristic of host shifts [59]. Indeed, examples of bacterial host shifts in which the ancestral (donor) host has been identified are largely limited to the jumps of *S. aureus* between livestock and humans, the shift of *M. gallisepticum* from livestock into wildlife and the shift of *Helicobacter pylori* from humans to large felines [59]. Although we have suggested three hypotheses to explain the lower likelihood of bacterial pathogens to evolve specialization in novel hosts, we cannot rule out the possibility that the reduced number of bacterial host shifts also stems, in part, from a taxonomical artefact. Indeed, while many bacterial ‘species’ display a broad host range and are considered multi-host parasites [51,60], there is growing evidence that individual clones, strains or populations of these species are, in fact, host-specific and show little evidence of host sharing [61]. Comparative studies considering bacteria by their species names may, as a result, underestimate the number of distinct lineages in circulation [62], and therefore of host shift events. Either way, using a modelling approach, we show that the ability of bacterial pathogens to routinely deal with environmental variation, as well as their mode and tempo of molecular evolution are, in any case, likely to reduce their propensity for host specialization. Bacterial emergence may therefore be more likely to consist of transient spillovers or of increased host range, depending on the bacterial pathogen's ability to adapt to the novel host. Whether and how differences in the propensity to specialize on the novel host affect epidemiological dynamics or the preventative measures that can be put in place to prevent bacterial outbreaks, remain to be determined.

## Supplementary Material

Supplement information

## Data accessibility

The computer code to generate the stochastic simulation model can be downloaded from github: https://github.com/megabyte22/pathogen_specialization.
