# Supplementary material for: Understanding the emergence of bacterial pathogens in novel hosts
Source: Philos Trans R Soc Lond B Biol Sci. 2019 Aug 12;374(1782):20180328. doi: 10.1098/rstb.2018.0328 (PMC6711297; doi:10.1098/rstb.2018.0328)
Supplement: Supplement information [file rstb20180328supp1.pdf]

Understanding the emergence of bacterial pathogens in novel hosts

Camille Bonneaud, Lucy A. Weinert and Bram Kuijper

Online Supplement

S1 Host specialization in the face of phenotypic plasticity: analytical model

S1.1 Evolutionary dynamics

To calculate the evolutionary change in v, we consider eqns. (3,4) in the main text, which reflect the change in infected hosts. We write both equations as

$$\frac{\mathrm{d}\mathbf{N}}{\mathrm{d}t} = \mathbf{m}\mathbf{N}, \tag{S1}$$

where $\mathbf{N} = [I_\mathrm{a}, I_\mathrm{n}]$ and the matrix $\mathbf{m} = \mathbf{B} - \mathbf{D}$ reflects the difference between birth and death rates: elements of $\mathbf{B}$ are given by $\beta_{ji} x_i$ and $\mathbf{D}$ is a diagonal matrix whose elements are $\alpha_i + \delta_i + \gamma_i$.

As in [1], we can then use a standard result (e.g., [2] chapters 10 and 12) to calculate the selection gradient $\mathcal{V}$ in virulence trait v:

$$\mathcal{V} = \sum_{i \in \{\mathrm{a},\mathrm{n}\}} \sum_{j \in \{\mathrm{a},\mathrm{n}\}} y_i x_j \left. \frac{\partial m_{ij}}{\partial \hat{v}} \right|_{\hat{v}=v}, \tag{S2}$$

where y_i is the reproductive value of a parasite in host i and x_j the stable class frequency of host j. These can be easily calculated as left and right dominant eigenvectors of $\mathbf{m}$ and are not given here. Evolutionary change Δv in virulence is then given by (e.g., [3, 4]):

$$\Delta v = C \cdot \mathcal{V}, \tag{S3}$$

where C is a constant reflecting the amount of heritable genetic variation on which natural selection can act. We then iterate eq. (S3) numerically until we find the candidate evolutionarily stable virulence $v^\star$ where $\Delta v = 0$, which is by definition the convergence stable virulence [5].

S1.2 Calculating branching points

By numerically iterating eq. (S3), we can find the candidate evolutionarily stable strategies (sensu [6]). By inspecting higher order derivatives with respect to mutant virulence $\hat{v}$, allowing us to assess that the candidate evolutionarily stable virulence strategy is indeed evolutionarily stable (i.e., an ESS: [6]), or whether mutants that either have higher or lower values of virulence can

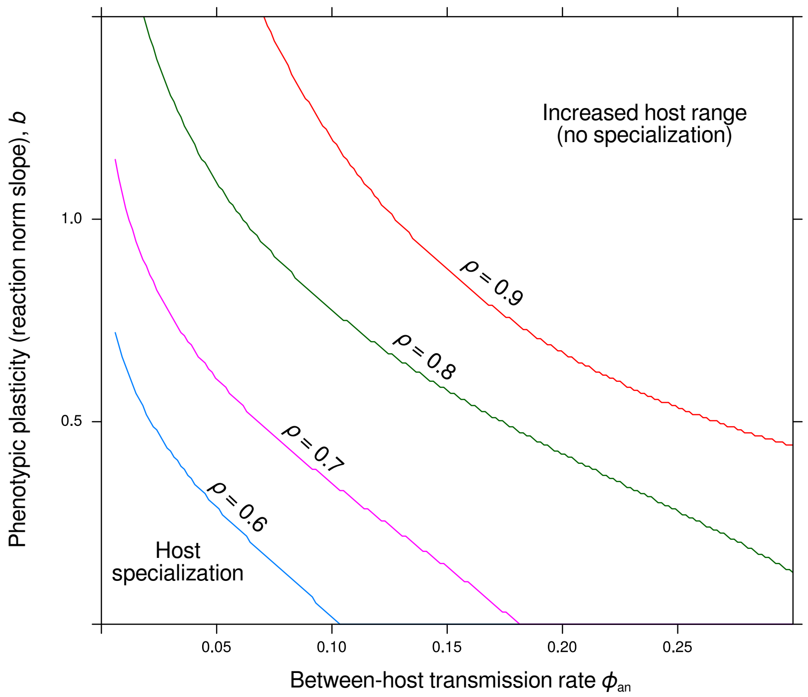

Figure S1: Area to the left of each line depicts the occurrence of host specialization of virulence, whereas the area to the right of each curve reflects an increased host range. Increasing values of ρ reflect increasing differences between ancestral and novel hosts in how virulence affects pathogen propagule production versus host mortality (see main text). Host shifts only occur when values of phenotypic plasticity b are low and between-species transmission rates of parasites ϕ_{an} are low. Parameters: $p = 0.5, \lambda = 2000$, $\delta_{\mathrm{a}} = \delta_{\mathrm{n}} = 1.0$, $\tau = 2.0$, $\gamma_{\mathrm{a}} = \gamma_{\mathrm{n}} = 0$, $\phi_{\mathrm{aa}} = \phi_{\mathrm{nn}} = 1$, $\phi_{\mathrm{an}} = \phi_{\mathrm{na}}$.

invade. In the latter case, evolutionary branching occurs, resulting in a specialization of virulence in either host. In a physiologically structured population, branching occurs when (Ido Pen, personal communication):

$$0 < \left[\mathbf{y}^{\mathrm{T}}\mathbf{m}''\mathbf{x} - 2\mathbf{y}^{\mathrm{T}}\mathbf{m}\left(\mathbf{m} - \lambda\mathbf{I} + \mathbf{x}^{\mathrm{T}}\mathbf{x}\right)^{-1}\mathbf{m}'\mathbf{x}\right]\Big|_{\hat{v}=v}, \tag{S4}$$

where primes denote derivatives with respect to mutant virulence $\hat{v}$. Figure S1 highlights for which conditions specialization of virulence occurs, showing that high values of pre-existing plasticity and high contact rates between different host species hamper the occurrence of host shifts.

S2 Gillespie simulations

The analytical model in eqns (1-5) int the main text only allows us to calculate when and where evolutionary branching occurs, but does not allow us to assess the resulting degree of pathogen specialization. To overcome this, we have developed stochastic Gillespie simulations. The C++ source code is available at https://github.com/megabyte22/pathogen_specialization.

In contrast to the analytical model, we assume that three unlinked haploid loci evolve: (i) the genetic value a underlying virulence, (ii) reaction norm slope b and (iii) the rate of recombination

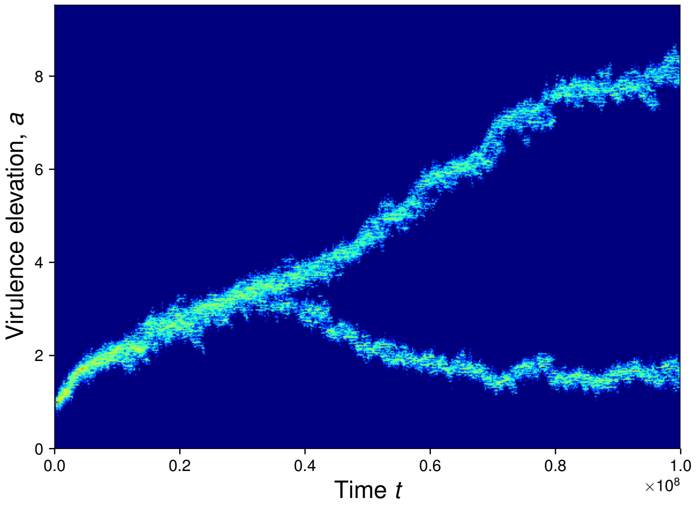

Figure S2: The evolution of a host shift through virulence specialization.

h. Recombination implies that a pathogen replaces its own allele with that from a randomly chosen pathogen in the same host population (mimicking horizontal transmission), where recombination can affect a or b alleles with equal probability. Loci mutate using a continuum of alleles model [7], with mutation rate μ_a (see Figure 1), $\mu_b = 0.01$ and $\mu_h = 0$ (Figure 1A-C) or $\mu_h = 0.01$ (Figure 1D-F). Upon mutation, allelic values are updated with a random number drawn from a uniform distribution between -0.1 and 0.1. An example of evolutionary branching of virulence is given in Figure S2.

S2.1 Parameters Figure 1

The parameter values used in the stochastic simulations of Figure 1 in the main text are: $\gamma_{\text{a}} = \gamma_{\text{n}} = 0$, $\delta_{\text{a}} = \delta_{\text{n}} = 1$, $\phi_{\text{aa}} = \phi_{\text{nn}} = 1$, $p = 0.5$, $\rho = 0.9$, $\phi_{\text{an}} = \phi_{\text{na}}$ (see values in Figure 1, main text). Initial population sizes: $S_{\text{a}}(t = 0) = 1000$, $S_{\text{n}}(t = 0) = 2000$, $I_{\text{a}}(t = 0) = 1000$, $I_{\text{n}}(t = 0) = 0$. Simulations ran for 1×10^8 timesteps.

References

[1] Gandon, S., 2004 Evolution of multihost parasites. *Evolution* **58**, 455–469. doi:10.1111/j.0014-3820.2004.tb01669.x.

[2] Otto, S. P. & Day, T., 2007 *A Biologist's Guide to Mathematical Modeling in Ecology and Evolution*. Princeton: Princeton University Press.

[3] Taylor, P. D., 1996 Inclusive fitness arguments in genetic models of behaviour. *J. Math. Biol.* **34**, 654–674. doi:10.1007/bf02409753.

[4] Dieckmann, U. & Law, R., 1996 The dynamical theory of coevolution: a derivation from stochastic ecological processes. *J. Math. Biol.* **34**, 579–612. doi:10.1007/BF02409751.

[5] Christiansen, F. B., 1991 On conditions for evolutionary stability for a continuously varying character. *Am. Nat.* **138**, 37–50. doi:10.1086/285203.

[6] Geritz, S. A. H., Kisdi, É., Meszéna, G. & Metz, J. A. J., 1998 Evolutionarily singular strategies and the adaptive growth and branching of the evolutionary tree. *Evol. Ecol.* **12**, 35–57. doi:10.1023/a:1006554906681.

[7] Kimura, M., 1965 A stochastic model concerning the maintenance of genetic variability in quantitative characters. *Proc. Natl. Acad. Sci. USA*. **54**, 731–736. doi:10.1073/pnas.54.3.731.
